# Breathlessness Predicts Mortality in Adults: A Systematic Review and Meta-Analysis

**DOI:** 10.7759/cureus.39192

**Published:** 2023-05-18

**Authors:** Dheeraj K Sethi, James Rhodes, Rebecca Ferris, Radhika Banka, Allan Clarke, Eleanor K Mishra

**Affiliations:** 1 Norwich Medical School, University of East Anglia, Norwich, GBR; 2 Respiratory Medicine, Norfolk and Norwich University Hospital, Norwich, GBR; 3 Respiratory Medicine, P. D. Hinduja National Hospital & Medical Research Centre, Mumbai, IND

**Keywords:** mmrc dyspnoea scale, mmrc, predictor of survival, systematic review and meta-analysis, breathlessness, dyspnoea, modified mrc scale

## Abstract

Breathlessness is a commonly encountered symptom, and although its relationship with mortality is well established for many conditions, less clear is this relationship in healthy adults. This systematic review and meta-analysis examines whether breathlessness is associated with mortality in a general population. This is important in understanding the impact of this common symptom on a patient's prognosis. This review was registered with PROSPERO (CRD42023394104). Medline, EMBASE, CINAHL and EMCARE were searched for the terms ‘breathlessness’ and ‘survival’ or ‘mortality’ on January 24, 2023. Longitudinal studies of >1,000 healthy adults comparing mortality between breathless and non-breathless controls were eligible for inclusion. If an estimate of effect size was provided, studies were included in the meta-analysis. Eligible studies underwent critical appraisal, data extraction and risk of bias assessment. A pooled effect size was estimated for the relationship between the presence of breathlessness and mortality and levels of severity of breathlessness and mortality. Of 1,993 studies identified, 21 were eligible for inclusion in the systematic review and 19 for the meta-analysis. Studies were of good quality with a low risk of bias, and the majority controlled for important confounders. Most studies identified a significant relationship between the presence of breathlessness and increased mortality. A pooled effect size was estimated, with the presence of breathlessness increasing the risk of mortality by 43% (risk ratio (RR): 1.43, 95% confidence interval (CI): 1.28-1.61). As breathlessness severity increased from mild to severe, mortality increased by 30% (RR: 1.30, 95% CI: 1.21-1.38) and 103%, respectively (RR: 2.03, 95% CI: 1.75-2.35). The same trend was seen when breathlessness was measured using the modified Medical Research Council (mMRC) Dyspnoea Scale: mMRC grade 1 conferred a 26% increased mortality risk (RR: 1.26, 95% CI: 1.16-1.37) compared with 155% for grade 4 (RR: 2.55, 95% CI: 1.86-3.50). We conclude that mortality is associated with the presence of breathlessness and its severity. The mechanism underlying this is unclear and may reflect the ubiquity of breathlessness as a symptom of many diseases.

## Introduction and background

Background

Breathlessness is the subjective experience of breathing discomfort [1]. It is a common symptom in a wide range of conditions, including cardiovascular disease (e.g. cardiac failure), respiratory disease (e.g. chronic obstructive pulmonary disease (COPD), interstitial lung disease) and cancer. The relationship between breathlessness and mortality has been studied in many populations with chronic diseases. In patients with idiopathic pulmonary fibrosis, breathlessness assessed using the modified Medical Research Council (mMRC) chronic dyspnoea score is associated with poor survival [2-4]. In patients presenting with acute congestive cardiac failure, subacute breathlessness is predictive of increased one-year mortality [5]. Cancer patients presenting to the emergency department with breathlessness have a mean survival of only 12 weeks [6].

A similar relationship between breathlessness and mortality has been observed in population-based, longitudinal studies of healthy individuals. A systematic review published by Pesola and Ahsan in 2016 included 10 prospective longitudinal studies, all of which demonstrated breathlessness is an independent predictor of mortality [7]. A meta-analysis was not performed, and the strength of the association between breathlessness and mortality was not described. Additionally, the association between levels of breathlessness severity and mortality was not studied in detail.

The value of breathlessness in predicting mortality is demonstrated by its inclusion in predictive scores of mortality in individual patients. The BODE index, a validated prognostic score for patients with COPD, includes breathlessness as well as body mass index, airflow obstruction and exercise capacity [8]. Similarly, the Palliative Prognostic Index includes breathlessness as one element of a validated score to predict prognosis in patients with advanced cancer [9].

Breathlessness can be assessed in a variety of ways. The simplest way is to ask patients whether they are breathless, yielding a binary ‘yes’ or ‘no’ response. Responses can be subjective, where patients and studies may employ different cut-offs to distinguish between a positive or negative answer. The introduction of intermediate levels of breathlessness, by categorising breathlessness as ‘mild’, ‘moderate’ or severe’, does help stratify breathlessness severity, but similarly suffers from ambiguous boundaries between these grades. Validated tools, such as the mMRC Dyspnoea Scale, can be used to better assess breathlessness. They allow clinicians to stratify levels of breathlessness severity, by providing examples of real-world activities that may evoke breathlessness, lending more objectivity and consistency to the measurement of breathlessness. The objective of this systematic review was to examine whether breathlessness is associated with increased mortality in the general population. We also performed a meta-analysis to quantify the strength of this relationship, both for the presence or absence of breathlessness and for increasing levels of severity.

## Review

Methods

The study was registered with PROSPERO (CRD42023394104). A systematic review was conducted of published studies in adults. The exposure of interest was self-reported breathlessness, compared to subjects who did not report breathlessness. The outcome of interest was all-cause mortality. There were no restrictions on the date range, location of the study or length of follow-up. Only English-language articles were included. Review articles, conference abstracts, case series, case reports and studies which were not empirical research were excluded. Retrospective or prospective studies of at least 1,000 participants were felt to be adequately powered to detect a relationship between mortality and breathlessness. Medline, EMBASE, CINAHL and EMCARE were searched for the terms ‘breathlessness’ and ‘survival’ or ‘mortality’ on January 24, 2023. Our full search strategy is available in Appendix 1.

After the removal of duplicate references, the titles and abstracts of all papers retrieved from the searches were screened for eligibility by independent reviewers. Full texts were retrieved for any papers fulfilling these criteria. The full texts were screened independently by two reviewers against the same inclusion and exclusion criteria. Disagreements at either stage of screening were resolved by a third independent reviewer.

All papers were critically appraised for quality and risk of bias using the Joanna Briggs Institute Critical Appraisal tool [10], and poor quality studies (as assessed by both reviewers independently) were excluded from the analysis. Data was extracted by both reviewers onto a Microsoft Excel spreadsheet (Microsoft, Washington, US). Information collected from each paper included the type of study, number of participants, measure of breathlessness, length of follow-up, whether regression analysis was performed to account for confounding variables, risk of bias, data analysis methods, whether any relationship was found between breathlessness and mortality and how this relationship was reported. Authors were contacted to provide additional data if needed; otherwise, the figures available in the publication or supplementary materials were used.

Studies were eligible for inclusion in the meta-analysis if they reported relative risk (RR) or hazard ratio (HR) describing the relationship between breathlessness and mortality. Those reporting an odds ratio (OR) were eligible if sufficient information was included to calculate an RR [11]. A pooled estimate of the RR was estimated by a random effects model due to significant between-study heterogeneity. Heterogeneity was estimated using the Higgins *I*^2^ statistic. All analyses were completed using RevMan 5.4.1 (September 20, Cochrane Collaboration, London, UK).

The meta-analysis was performed using the results that maximally adjusted for potential confounders. Where studies only provided unadjusted results, these were included in the meta-analysis. Effect measures describing the effect for the whole cohort were used if available. Where studies reported on subgroups individually (i.e. males, females), each subgroup was included as a separate study. Publication bias was investigated by visual assessment of inverted funnel plots. Where mortality was assessed at multiple time points (i.e. one-day, seven-day, one-year mortality), the longest follow-up period was used.

Owing to the heterogeneity in defining breathlessness, separate meta-analyses were performed for different levels of breathlessness versus no breathlessness/mMRC grade 0. These were as follows: ‘mild/mMRC grade 1’ breathlessness, ‘severe’ breathlessness/mMRC grade 4 and each individual mMRC grade of breathlessness (i.e. grade 1/2/3/4). Where studies reported three levels of severity of breathlessness, their lowest severity was eligible for inclusion in the mild subgroup and their most severe level was eligible for the severe subgroup.

A sensitivity analysis was performed to assess the impact of select study characteristics. This included repeating the meta-analysis using an unadjusted or minimally adjusted value in place of the maximally adjusted value. Additionally, the studies that only reported an HR were analysed, excluding those reporting an RR. We also employed a fixed effects, rather than a random effects, model to see if this altered the significance of our findings.

Results

**Figure 1:**
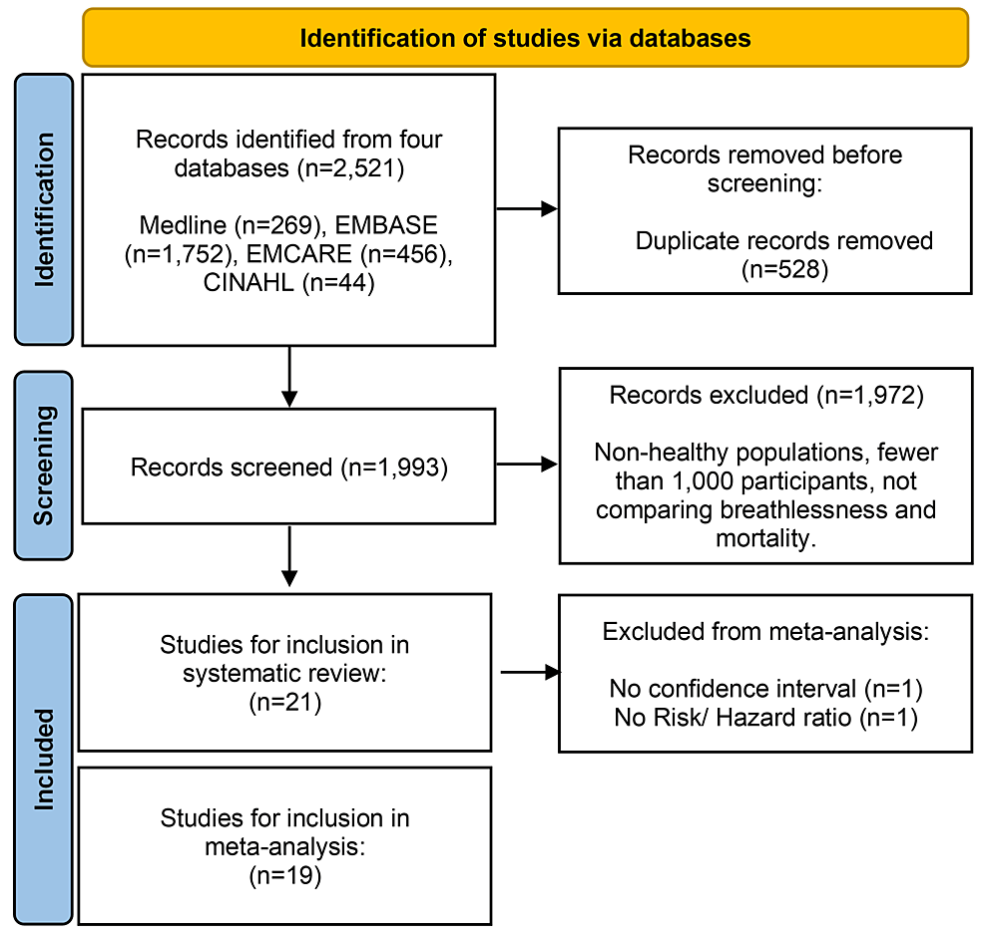
PRISMA flow diagram PRISMA: Preferred Reporting Items for Systematic Reviews and Meta-Analyses.

Our search yielded 2,521 records, but after deduplication, a total of 1,993 abstracts were identified. Of these, 253 were selected for whole paper review, with 21 eligible. The main reasons the studies were excluded were that they included fewer than 1,000 participants and did not report a relationship between breathlessness and mortality or studied an inappropriate population (i.e. comorbid or non-healthy populations). Our search strategy is summarised in a Preferred Reporting Items for Systematic Reviews and Meta-Analyses (PRISMA) flow diagram (Figure 1).

Eligible studies undergoing critical appraisal were all of high quality, with little risk of bias. Common sources of bias included loss to follow-up and rates of missing data not being stated. Missing data was commonly dealt with by excluding incomplete datasets, although few studies performed a sensitivity analysis.

Across 21 eligible studies, 357,372 participants were included (median: 7,360, interquartile range: 2,792-12,501). One study was a retrospective cohort study [12], and the remaining 20 were prospective cohort studies. All studies were published between 1985 and 2021. Follow-up duration was not uniform in how it was described, due to the variety of different study designs in use. Some studies reported the mean or median follow-up duration, whilst others described the amount of time patients were prospectively followed up. The shortest period of follow-up was two years, and the longest was 31 years [12,13]. Study characteristics are summarised in Table 1.

**Table 1:** Characteristics of 21 studies included the systematic review and meta-analysis mMRC: modified Medical Research Council, OR: odds ratio, RR: risk ratio, HR: hazard ratio, CI: confidence interval. *Confidence intervals not provided, hence ineligible for meta-analysis. **Insufficient data provided to calculate RR from OR, hence ineligible for meta-analysis. ***Data from external communications with authors.

Study, location, design	Number of participants	Breathlessness measure	Follow-up	Mortality risk (95% CI)
Ahmed et al. (2012) [14], Wales, prospective	1,169	Binary, mMRC ≥3 OR	10 y	1.94 (1.11-3.38)
Berraho et al. (2013) [15], France, prospective	3,646	mMRC 1, 2,3+4 HR	13 y	mMRC 1: 1.13 (1.01-1.26), mMRC 2: 1.42 (1.25-1.63), mMRC 3+4: 1.90 (1.61-2.25)
Carpenter et al. (1989) [16], UK, prospective	1,532	mMRC 1-4 RR	27 y	mMRC 1: 1.5 (1.2-1.9), mMRC 3: 1.7 (1.1-2.5), mMRC 4: 2.0 (1.2-3.2), mMRC 5: 3.6 (2.1-6.0)
Edjolo et al. (2013) [17], France, prospective	2,517	Binary HR	20 y	Male 1.23 (1.06-1.42), Female 1.20 (1.07-1.36)
Feng et al. (2022) [13], USA, prospective	4,621	Binary HR	31 y	1.57 (1.16-2.12)
Figarska et al. (2012) [18], Netherlands, prospective	7,360	mMRC 2-3, mMRC 4 HR	43y	mMRC 2-3: 1.5 (1.3-1.7), mMRC 4: 1.9 (1.4-2.5)
Frostad et al. (2006) [19], Norway, prospective	17,678	Ordinal HR	30 y	Male mild: 1.52 (1.37-1.70), Male severe: 2.14 (1.84-2.48), Female mild: 1.34 (1.21-1.50), Female severe: 1.40 (1.19-1.64)
Gulsvik et al. (2020) [20], Norway, prospective	158,702	mMRC 1-4 HR	27 y	mMRC 1: 1.28 (1.24-1.32), mMRC 2: 1.52(1.47-1.57), mMRC 3: 1.90 (1.80-2.00), mMRC 4: 1.78 (1.63-1.95)
Kaplan and Kotler (1985) [21], USA, prospective*	4,590	Binary RR	9 y	Male 1.61 and 2.35, Female 1.32 and 1.83
Kim et al. (2016) [22], USA, prospective	48,914	Binary HR	11 y	1.12 (1.05-1.2)
Knuiman et al. (1999) [23], Australia, prospective	4,277	Binary, mMRC ≥3 HR	26 y	Male: 1.832 (1.335-2.514), Female: 1.742 (1.295-2.342)
Leivseth et al. (2014) [24], Norway, Prospective	10,491	Ordinal: stratified by activity HR	14 y	Male uphill: 1.34 (0.83-2.15), Male sitting: 1.94 (0.92-4.09), Female uphill: 1.02 (0.70-1.47), Female sitting: 1.22 (0.68-2.17)
Pan et al. (2019) [25], China, prospective	16,777	Binary, mMRC ≥3 HR	11 y	1.36 (1.17–1.59)
Pesola et al. (2016) [26], Bangladesh, prospective	11,533	Binary HR	11 y	2.10 (1.74-2.52)
Petrie et al. (2020) [27] Australia, prospective***	2,087	Binary HR	22 y	1.30 (1.18-1.44)
Roberts et al. (2013) [12], USA, retrospective**	12,501	Binary OR	2 y	2.2 (1.9-2.6)
Rozanski et al. (2014) [28], USA, prospective	12,232	Binary HR	11 y	1.53 (1.2-2.0)
Santos et al. (2016) [29], USA, prospective	10,881	Ordinal HR	19 y	Mild: 1.16 (1.06-1.26), Severe: 1.96 (1.55-2.48)
Stavem et al. (2006) [30], Norway, prospective	1,623	mMRC 1, mMRC 2-4 RR	26 y	mMRC 1: 1.77 (1.40-2.23), mMRC 2-4: 2.02 (1.21-3.39)
Tessier et al. (2001) [31], France, prospective	2,792	mMRC 1-4 RR	8 y	mMRC 1: 1.15 (0.98-1.36), mMRC 2: 1.4 (1.2-1.7), mMRC 3: 2.01 (1.6-2.5), mMRC 4: 6 (3.7-9.7)
Waller et al. (2014) [32], Finland, prospective	21,379	mMRC 1-4 HR	28 y	mMRC 1: 1.17 (1.09-1.26), mMRC 2: 1.5 (1.34-1.68), mMRC 3: 1.95 (1.65-2.30), mMRC 4: 2.15 (1.74-2.66)

The mMRC Dyspnoea Scale was used in some capacity in many studies. Four studies used an mMRC grade of ≥3 to identify the presence of breathlessness. Six studies reported the relationship between mortality and individual mMRC grades, but in some instances, grades were combined and not all individual grades were reported. Four studies categorised breathlessness as mild, moderate or severe, using the mMRC Dyspnoea Scale, although different cut-offs for each level of severity were used.

Systematic review

There was significant heterogeneity in how breathlessness was defined across studies. Breathlessness was described as a binary variable in 11 studies, which compared mortality with the presence or absence of breathlessness. Three of these studies reported their findings for males and females separately and did not provide an overall estimate [17,21,23]. One of these studies found a significant relationship between breathlessness in males but not in females [21], whilst all other studies found a significant relationship.

Ten studies reported breathlessness as an ordinal variable and described the relationship between various levels of breathlessness severity and mortality. One study stratified severity by activities that were likely to elicit breathlessness. This study reported results for males and females separately and identified breathlessness while walking was associated with mortality for males and females, but interestingly, breathlessness while sitting was not [24]. The remaining nine studies that looked at breathlessness severity found it was positively related to mortality.

Meta-analysis

**Figure 2:**
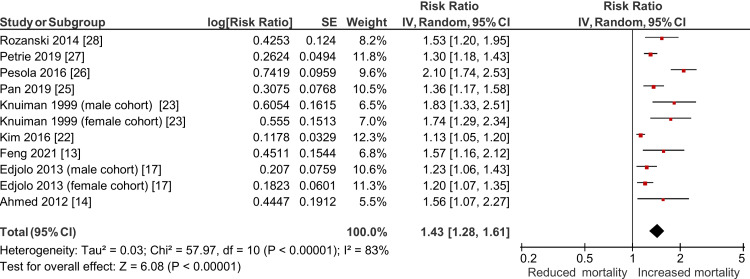
Forest plot demonstrating the relationship between the presence or absence of breathlessness and mortality, estimated using a random effects model (n=11, nine studies)

An HR was provided in 16/21 studies, an RR in three and an OR in two, one of which provided sufficient information to calculate an RR [14]. One study reporting an HR was ineligible for inclusion in the meta-analysis as they did not report a confidence interval [21]. Consequently, a total of 19 studies were eligible for inclusion in the meta-analysis. Of nine studies looking at breathlessness as a binary variable and eligible for inclusion in the meta-analysis, we estimated a pooled effect size of RR 1.43, with a 95% confidence interval (CI): 1.28-1.61, *I*^2^ = 83% (Table 2, Figure 2). Visual inspection of funnel plots (Appendix 2) did not suggest significant publication bias.

**Table 2:** Pooled estimates of relative risk for each subgroup, estimated using a random effects model mMRC: modified Medical Research Council. Where a study reported results for males and females separately, each cohort was entered as a separate study.

Subgroup	Pooled effect ratio
Breathless vs non-breathless (n=11, 9 studies); Heterogeneity: Tau² = 0.03; Chi² = 57.97, df = 10 (P < 0.00001); *I*² = 83%	1.43 (1.28-1.61)
Mildly breathless (including mMRC 1) vs non-breathless (n=12, 10 studies); Heterogeneity: Tau² = 0.01; Chi² = 43.05, df = 11 (P < 0.0001); *I*² = 74%	1.30 (1.21-1.38)
Severely breathless (including mMRC 4) vs non-breathless (n=12, 10 studies); Heterogeneity: Tau² = 0.04; Chi² = 49.04, df = 11 (P < 0.00001); *I*² = 78%	2.03 (1.75-2.35)
mMRC grade 1 vs grade 0 (n=6); Heterogeneity: Tau² = 0.01; Chi² = 20.51, df = 5 (P = 0.001); *I*² = 76%	1.26 (1.16-1.37)
mMRC grade 2 vs grade 0 (n=5); Heterogeneity: Tau² = 0.00; Chi² = 1.27, df = 4 (P = 0.87); *I*² = 0%	1.51 (1.47-1.56)
mMRC grade 3 vs grade 0 (n=4); Heterogeneity: Tau² = 0.00; Chi² = 0.32, df = 3 (P = 0.96); *I*² = 0%	1.91 (1.82-2.01)
mMRC grade 4 vs grade 0 (n=5); Heterogeneity: Tau² = 0.10; Chi² = 30.91, df = 4 (P < 0.00001); *I*² = 87%	2.55 (1.86-3.50)

**Figure 3:**
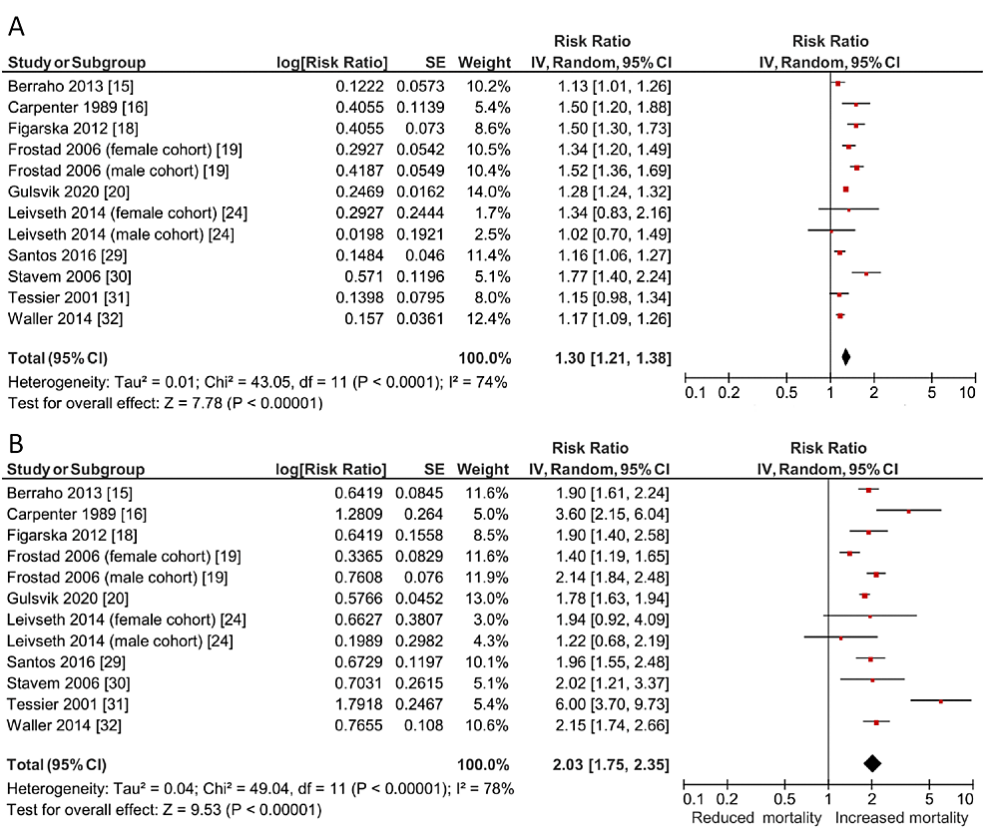
Forest plot demonstrating the relationship between increasing breathlessness severity and mortality, estimated using a random effects model mMRC: modified Medical Research Council. Subgroups include those with a) mild breathlessness (including mMRC grade 1) and b) severe breathlessness (including mMRC grade 4).

**Figure 4:**
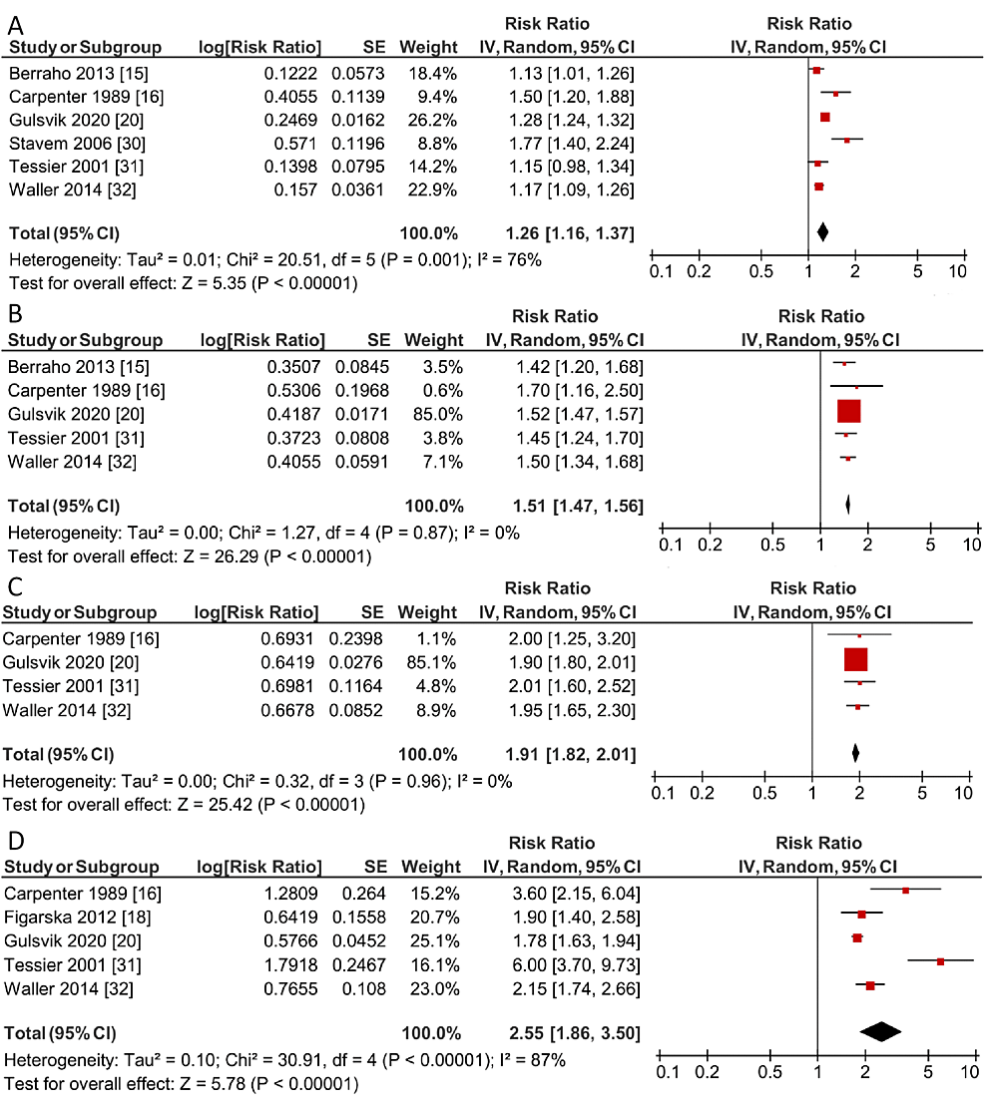
Forest plot demonstrating the relationship between the increasing breathlessness severity and mortality, estimated using a random effects model mMRC: modified Medical Research Council. Subgroups include those with a) mMRC grade 1, b) mMRC grade 2, c) mMRC grade 3, and d) mMRC grade 4.

Subgroups compared with non-breathless controls included mildly breathless (including mMRC grade 1), severely breathless (including mMRC grade 4) and individual grades of breathlessness according to the mMRC Dyspnoea Scale. Meta-analyses for each above group showed a positive, significant relationship between increasing breathlessness severity and mortality, as demonstrated in Table 2 and Figures 3-4. Overall, the analysis showed that mildly breathless participants have a 30% increased risk of mortality (RR: 1.30, 95% CI: 1.21-1.38, *I*^2^ = 74%), whereas the severely breathless had a 103% increased risk of mortality (RR: 2.03, 95% CI: 1.75-2.35, *I*^2^ = 78%).

Similarly, ascending grades of breathlessness, as stratified by mMRC grades, were also associated with increased risk of mortality: mMRC grade 1 had an RR of 1.26 (95% CI: 1.16-1.37, *I*^2^ = 76%), mMRC grade 2 had an RR of 1.51 (95% CI: 1.47-1.56, *I*^2^ = 0%), mMRC grade 3 had an RR of 1.91 (95% CI: 1.82-2.01, *I*^2^ = 0%) and mMRC grade 4 had an RR of 2.55 (95% CI: 1.86-3.50, *I*^2^ = 87%), as shown in Figure 4. After excluding the retrospective study and the three studies reporting an RR in turn, our findings remained significant. Additionally, after substituting the effect sizes with unadjusted or minimally adjusted values in place of the maximally adjusted models used initially, our findings remained significant across all groups and levels of breathlessness. After changing our underlying assumptions and switching our method of analysis to a fixed effects model from a random effects model, there were no significant changes to our findings, and the trends described above remained valid. Consequently, our sensitivity analysis confirmed the robustness and significance of our findings. The meta-analysis established a positive relationship between the presence of breathlessness and mortality, and that increasing breathlessness severity was associated with a greater risk of death.

Discussion

Our results demonstrate that breathlessness is significantly associated with increased mortality in adults. The strength of this relationship is demonstrated both by the near-unanimous association between breathlessness and mortality across all studies and again by the increase in mortality risk with increasing breathlessness severity. Although this relationship has been clearly established above, the mechanism is not fully understood. The correlation may simply demonstrate the ubiquity of breathlessness as a symptom across a plethora of severe diseases. However, this review focussed on otherwise well patients, with most studies adjusting for significant confounders, such as age, gender, body mass index, lung function, smoking, etc. Consequently, breathlessness and mortality may be causally related. An explanation for this is that breathlessness initiates or perpetuates a negative spiral of deconditioning, loss of fitness and frailty, leading to increased mortality. Indeed, an intervention such as pulmonary rehabilitation, which is designed to relieve breathlessness and reverse deconditioning, improves survival in COPD patients [33,34].

The strengths of our work were that the studies included were of high quality and with a low risk of bias. The majority were prospective studies with over 1,000 participants, meaning that they were more likely to provide precise estimates of effect size. Additionally, most studies had long periods of follow-up. Visual inspection of our funnel plots suggested that our findings were not significantly impacted by publication bias, a common pitfall of systematic reviews and meta-analyses. This suggests we conducted a comprehensive and robust search.

Our work has some limitations. Many studies did not describe rates of missing data and how this was handled. Additionally, many studies did not state how breathlessness was assessed and few used a validated tool. Most studies asked patients whether they were breathless or not, but the exact questions used and how they were asked varied across studies. Some studies also graded breathlessness as ‘mild’, ‘moderate’ or ‘severe’, but again, with limited consistency in how these categories were defined, and with ambiguous boundaries between each level. Additionally, even where the mMRC Dyspnoea Scale was used, some studies defined an mMRC grade of greater than 2 as breathless, whilst others used a different cut-off or reported the relationship between mortality and breathlessness for each individual grade. Consequently, the variation in how studies categorised patients as breathless cannot be overlooked.

Furthermore, 19 out of 21 studies were performed in the Western world with the remaining two studies performed in China [25] and Bangladesh [26]. The lack of global representation suggests that our findings do not reflect the impact of regional epidemiology and may neglect the increased prevalence of infectious disease, variable nutritional status and the impact of air pollution in developing countries. Consequently, these findings should be interpreted contextually, and we recognise they may not be transferrable to patients who are breathless in countries where the disease burden is different to that conventionally seen in the Western world.

The subgroups with the least heterogeneity looked at individual grades of mMRC breathlessness and provided an upper and lower bound for breathless severity. Subgroups with higher levels of heterogeneity, by definition, did not have an upper limit of severity. This may represent the real-world variation in patients assigned the same breathlessness grading at the top or bottom of a scale. Additionally, the subgroup that studied the presence versus absence of breathlessness (*I*^2^=71%), without stratification by severity, also suffered from significant levels of heterogeneity. The source of this heterogeneity may be explained by inconsistent definitions of a breathless participant across studies. In one study, only the most severely breathless participants may be categorised as breathless, whilst another study may employ a lower threshold to define a breathless participant. To mitigate this, in future, we would recommend studies investigating breathlessness should explicitly describe how breathlessness has been assessed and utilised a more objective scale, such as the mMRC Dyspnoea Scale. Additional sources of heterogeneity include the comparison of hazard and risk ratios, differences in the duration of follow-up, the variation in populations studied and inconsistencies in which confounders had been controlled for across studies.

## Conclusions

Our findings have several implications. Studies of variables predictive of mortality or studies to create predictive scores should consider whether breathlessness is an important variable for their cohort. If so, breathlessness should be considered as an ordinal rather than binary variable, and tools such as the mMRC Dyspnoea Scale, which allows for its more precise assessment, should be used. Additionally, clinicians should be aware that breathless patients may be at higher risk of death, and quantifying patients’ breathlessness severity may aid prognostication. However, it is important to recognise that these results are from studies conducted in the Western world and neglects the local epidemiology seen in developing countries.

In summary, breathlessness is associated with mortality in adults. Our meta-analysis suggests that the presence of breathlessness increases the risk of mortality by 43% and breathlessness severity is similarly associated with increasing mortality. Increasing grades of breathlessness on the mMRC Dyspnoea Scale increase the risk of mortality from 26% for mMRC grade 1 to 155% for mMRC grade 4.

## Figures and Tables

**Figure 5:**
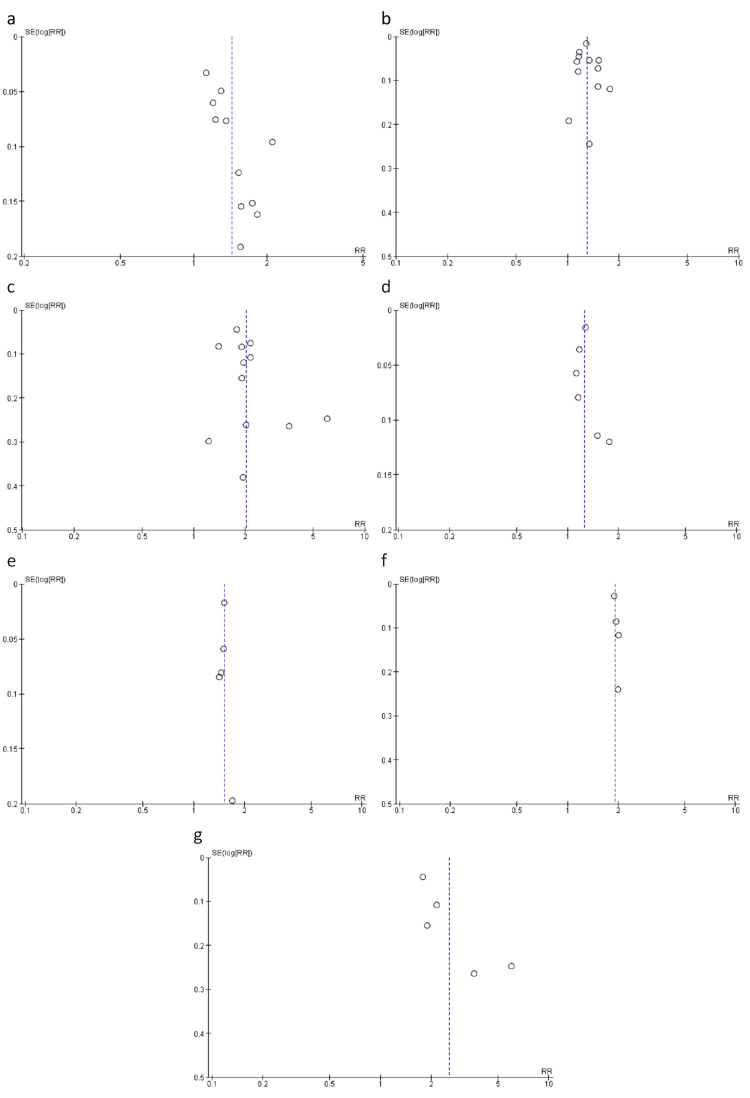
Funnel plots mMRC: modified Medical Research Council. Funnel plots for a) presence or absence of breathlessness, b) mild breathlessness (including mMRC grade 1), c) severe breathlessness (including mMRC grade 4), d) mMRC grade 1), e) mMRC grade 2, f) mMRC grade 3, and g) mMRC grade 4.

## Appendices

Appendix 1: Search strategy

1 Medline - exp DYSPNEA/ OR exp "DYSPNEA, PAROXYSMAL"/

2 Medline - (breathlessness).ti,ab

3 Medline - (1 OR 2)

4 Medline - exp SURVIVAL/

5 Medline - exp MORTALITY/

6 Medline - (4 OR 5)

7 Medline - (3 AND 6) [DT FROM 2019] [Human age groups Adult] [Languages English]

8 EMBASE - exp DYSPNEA/

9 EMBASE - (breathlessness).ti,ab

10 EMBASE - (8 OR 9)

11 EMBASE - exp *SURVIVAL/

12 EMBASE - exp *MORTALITY/

13 EMBASE - (11 OR 12)

14 EMBASE - (10 AND 13) [DT FROM 2019] [Languages English] [Human age groups Adult 18 to 64 years OR Aged 65+ years]

15 CINAHL -  exp DYSPNEA/ OR exp "DYSPNEA, PAROXYSMAL"/

16 CINAHL -  (breathlessness).ti,ab

17 CINAHL -  (15 OR 16)

18 CINAHL -  exp *SURVIVAL/

19 CINAHL -  exp *MORTALITY/

20 CINAHL -  (18 OR 19)

21 CINAHL -  (17 AND 20) [DT FROM 2019] [Human age groups All Adult] [Languages eng] 10

22 EMCARE - exp DYSPNEA/ 53109 23 EMCARE (breathlessness).ti,ab

24 EMCARE - (22 OR 23)

25 EMCARE - exp *SURVIVAL/

26 EMCARE - exp *MORTALITY/

27 EMCARE - (25 OR 26)

28 EMCARE  - (24 AND 27) [DT FROM 2019] [Languages English] [Human age groups Adult 18 to 64 years OR Aged 65+ years]

Appendix 2
